# Mixed-effects models for joint modeling of sequence data in longitudinal studies

**DOI:** 10.1186/1753-6561-8-S1-S92

**Published:** 2014-06-17

**Authors:** Yan Yan Wu, Laurent Briollais

**Affiliations:** 1 Samuel Lunenfeld Research Institute, Mount Sinai Hospital, 60 Murray Street, Toronto, Canada M5T 3L9

## Abstract

In this paper, we propose a novel mixed-effects model for longitudinal changes of systolic blood pressure (SBP) over time that can estimate the joint effect of multiple sequence variants on SBP after accounting for familial correlation and the time dependencies within individuals. First we carried out agenome-wide association study (GWAS) using chromosome 3 single-nucleotide polymorphisms(SNPs) to identify regions associated with SBP levels. In a second step, we examined the sequence data to fine-map additional variants in these regions. Four SNPs from two intergenic regions (*PLXNA1-TPRA1, BPESC1-PISTR1*) and one gene (*NLGN1*) were detected to be significantly associated with SBP after adjusting for multiple testing. These SNPs were used to capture the multilocus genotype diversity in the regions. The multilocus genotypes derived from these four variants were then treated as random effects in the mixed-effects model, and the corresponding confidence intervals (Cis) were built to assess the significance of the joint effect of the sequence variants on SBP. We found that multilocus genotypes (GG,TT,AG,GG), (GG,TT,GG,GG), and (GG,TT,AA,AG) are associated with higher SBPand (GG,CT,AA,AA), (AA,TT,AA,AA), and (AG,CT,AA,AG) are associated with lower SBP. The linear mixed-effects models provide a powerful tool for GWAS and the analysis of joint modeling of multilocus genotypes.

## Background

The Genetic Analysis Workshop 18 (GAW 18) data set [1] is drawn from the San Antonio Family Study with a total of 959 participants from 20 families, and it includes genome-wide association study (GWAS) and whole genome sequencing(WGS) data on all individuals. The participants had 1to 4systolic blood pressure (SBP) measurements. At each examination, current use of antihypertensive medications, hypertension diagnosis, and current tobacco smoking status were recorded. This study provides a unique resource for elucidating genetic factors associated with longitudinal SBP after accounting for heterogeneity between individuals and families.

The mixed-effects model was used to analyze the longitudinal GWAS data and to estimate the joint effects of multiple sequence variants while accounting for familial correlation and the time dependencies within individuals. A real phenotype data set, along with GWAS and dosage sequence data on chromosome 3 were used for the analysis. The advantage of the mixed-effects model is that it can be used to model joint effects of genetic variants through the use of random effects. We developed this model for our analysis of the sequence variants [2].Our results suggest that there is significant variability in the effect of SBP across these multilocus genotypes.

## Methods

### Data

Four new variables were generated from the phenotype data.We combined hypertension diagnosis and antihypertensive medications to a single variable HTNmed with three levels: HTN med (hypertension with treatment), HTN no med (hypertension with no treatment), and non-HTN to avoid singularity problem when we fitted the models with interaction terms. The time-varying variablesmoking status has been collapsed to one variable, including 671 nonsmokers, 161 smokers, and 93 others. We used visit.year as a time variable in whichthe first visit is defined as 1 and the follow-up visits are 1+ number of years between the first and the follow-up visit, accounting forunevenly spaced visits. The age at the first visit (AGE.1) was used as a contextual effect at individual level accounting for the variation of SBP level between individuals at the beginning of the study. The SNPs on chromosome 3 with call rate greater than90%, *p-*values less than 1 × 10^−6 ^in the tests for Hardy Weinberg equilibrium,and minor allele frequency (MAF) less than 1% were excluded from our analysis.

### Mixed-effects model for longitudinal genome-wide association study

The SBP measurementhas two nested levels of random effects; the first is family, and the second is individual.We write the repeated measures of SBP over time, the response vector at the innermost level of grouping, as ${y_{ij}}_{,}i=1,\ldots ,m,j=1,\ldots ,J_{i},$ where *m *is the number of families and $J_{i}$ is the number of individuals within the *i*th family. The length of the vector $y_{ij}$ (i.e, the number of SBP measurements for the *j*th individual in the *i*th family) is $n_{ij}$ Thus, the model formula can be written as

$$\begin{array}{c} y_{ij}=X_{ij}\beta +Z_{i}b_{i}+Z_{ij}b_{ij}+\varepsilon_{ij},i=1,\ldots ,m,j=1,\ldots ,J_{i},\\ b_{i}\sim N\left(0,\psi_{1}\right),b_{ij}\sim N\left(0,\psi_{2}\right),\varepsilon_{ij}\sim N\left(0,\sigma_{\varepsilon }^{2}R_{ij}\right), \end{array}$$

where $X_{ij}$ is the fixed-effect regressor matrix and *β *is the corresponding fixed-effect vector; $Z_{i}$ and $Z_{ij}$ are family-level and individual-level random effects regressor matrices;and $b_{i}$ and $b_{ij}$ are random effect vectors (including random intercepts and random slopes of visit.year)corresponding to $Z_{i}$ and $Z_{ij}$, respectively. $\Psi_{1}$ and $\Psi_{2}$ are the family-level and individual variance matrices of random effects [3]. A continuous autoregressive correlation structure of order one (denoted as $R_{ij}$) was used to account for the unevenly spaced and unbalanced visits. Four covariates HTNmed, smoking, SEX, AGE.1, and visit.year were used in the mixed-effects model. Backward elimination technique was used for model selection and led to the model with fixed effects

$$\begin{array}{ccc} E\left[y_{ij}\text{|}b_{i},b_{ij}\right] & =\beta_{0}+AGE.1_{ij}.\beta_{AGE.1}+(visit.year_{ij}*HTNmed_{ij}.\beta_{visit.year*HTNmed} & \\ & +\left(HTNmed_{ij}*smoking_{ij}*SEX_{ij}\right).\beta_{HTNmHTNmed*smoking*SEX}, \end{array}$$

and the random effects are the random intercepts and random slopes of visit.year on both family and individual levels. For simplicity, the interaction terms contain the lower-order variables. Then the GWAS analysis was carried out in whicheach SNP was considered as a fixed main effect with no interaction with age and visit.year in our models.

### Joint modeling of sequence data using multilocus genotype patterns as random effects

Characterizing the association between multiple SNPs and disease outcomes can offer some new insights into our understanding of disease etiology while providing tools for making individualized treatment decisions. However, this presents an analytic challenge because of the large number of SNPs and the complex interaction among them. For example, 80 parameters are needed to account for the interaction term of four SNPs using codominant model.

The use of multilocus genotypes as random effects provides an intrinsic solution to the problem of dimensionality. A simple dimension reduction technique termed *patterning *is described in the HIV literature [4,5] and involves assigning observations to the same groups when the corresponding multilocus genotypes are identical. The presence of gene-gene interaction effect can be estimated by the variance of random intercept. And the gene-environment interaction effect can be estimated by the variance of random slope for the environmental variable. The parameters associated with each multilocus group are known as empirical best linear predictors (eBLUP). Further dimension reduction techniques such as clustering have been described in [6] in whichthe individuals are assigned to similar genotype groups based on hierarchical or K-means clustering methods.

In this paper, the multilocus groups are defined simply as groups of individuals with identical multilocus genotypes. These multilocus genotype groups resulting from patterning can be thought of as random samples from the general population of genotypes. It is therefore natural to treat them as random effects in a mixed-effects model. Thus, the mixed-effects model for this joint modeling of multilocus genotype patterns is same as the models specified in the previous section except that the nested levels of random effects are multilocus genotype group and individual instead of family and individual. The prediction intervals of the random effects for the multilocus genotype groups can be obtained using the bootstrap method [7] to assess the significance of the multilocus genotype. A multilocus genotype with a prediction interval of random effects not containing zero means that the estimated SBP level for these groups is significantly different from the population average. Our motivation for the patterning is to understand whether the risk of having high SBP is associated with an individual's multilocus genotype as a single contributor. Furthermore, modeling the relationship between genotype combinations and phenotype will potentially capture information on how genes interact with each other.

### Power analyses

The parametric bootstrap method [7] was used to perform power analyses in whichrandom samples weredrawn from the fitted models. For the GWAS, the power for each selected SNP was calculated based on 2000 parametric bootstrap samples using Bonferroni corrected level of significance at 0.05/number of independent GWAS tests. Similarly, for the joint modeling of multilocus genotype patterns as random effects, we calculated the power of detecting significant variance of random effects on the level of multilocus genotype patterns at 0.05 level of significance using 2000 parametric bootstrap samples.

## Results

**Figure 1:**
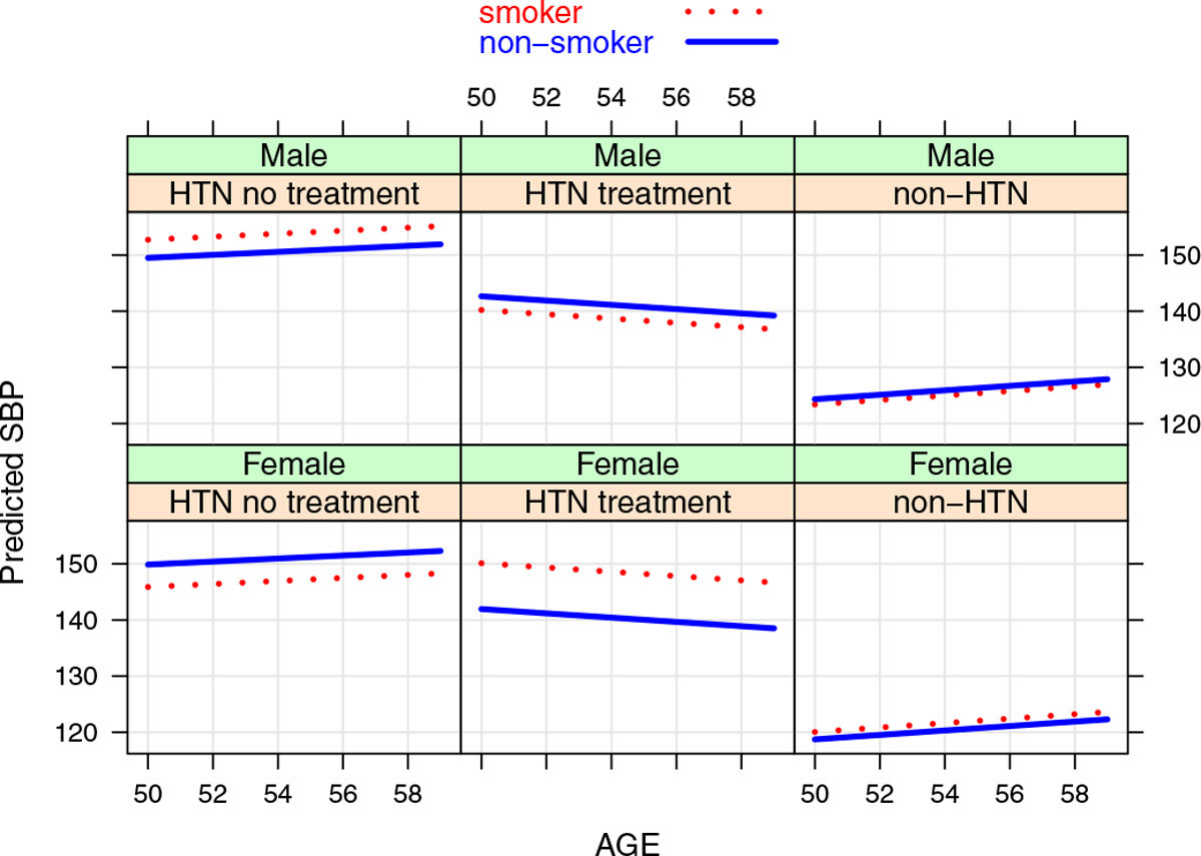
**Predicted systolic blood pressure (SBP) levels**.*HTN*, hypertension

A total of 59,649 SNPs and 860 individuals were used for the analysis.From the phenotype mixed-effects model, we found that for individuals who are on hypertension medication, the estimated SBP level for female smokers is approximately 8.2 mmHg(95% CI[0.98,15.80]) higher than that of female nonsmokers. The difference was not found in the other categories. Figure 1 displays the estimated SBP longitudinal profiles for men and women and smokers and nonsmokers with different hypertension statuses from age 50 to 60 years.

**Figure 2:**
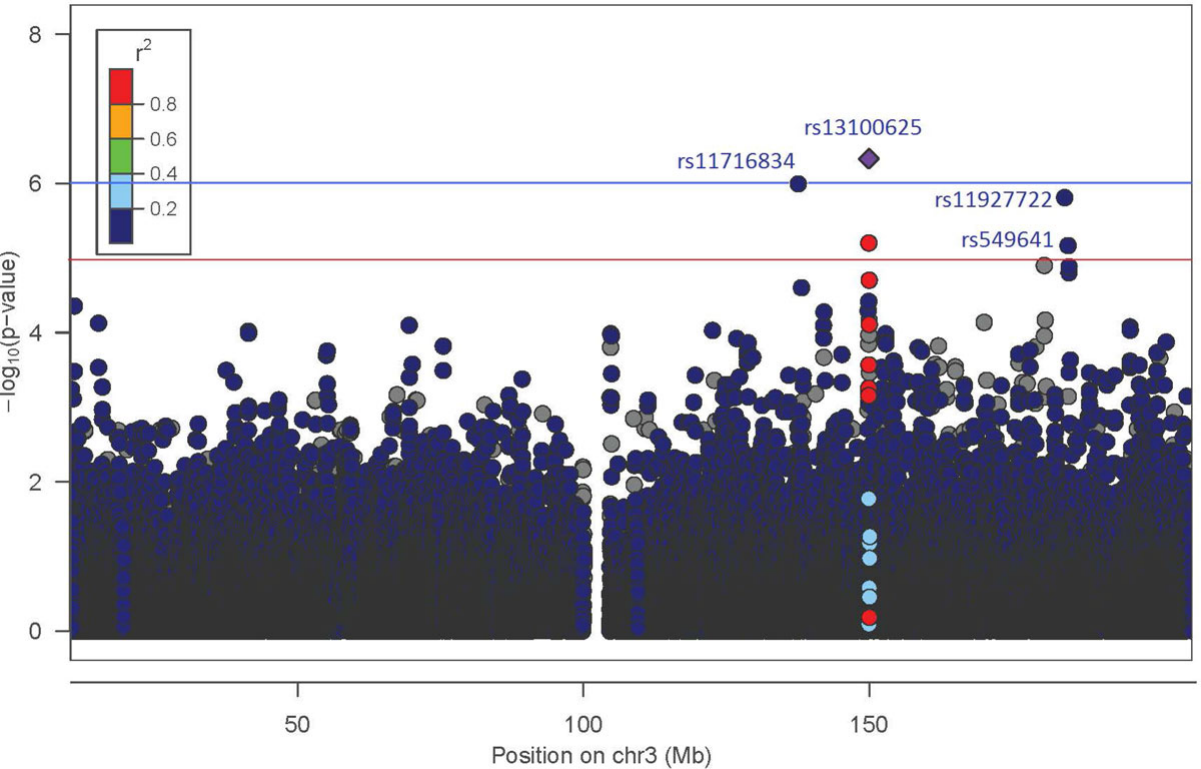
**Manhattan plot for genome-wide association study (GWAS) on chromosome 3**.

The GWAS on chromosome 3 has identified two intergenic regions,*PLXNA1-TPRA1*and *BPESC1-PISTR1*, and one gene,*NLGN1*, to be most significantly associated with SBP (Figure 2). Thus, we examined the sequence data to fine-map additional variants in these regions. We chose the SNPs that are most significantly associated with SBP for the joint modeling analysis. For SNPs that are in the same haplotype blocks, only one SNP with the lowest *p-*value was selected. Two SNPs from the two intergenic regions *PLXNA1-TPRA1 *and *BPESC1-PISTR1 *and two SNPs from the gene *NLGN1 *in different haplotype blocks were chosen for the patterning using a relaxed GWAS significance level $-log_{10}\left(p\right)=5$ (see Figure 2). Table 1 summarizes the information about the four SNPs and their associated gene names, MAFs,*p-*values, and powers calculated from the parametric bootstrap method.

**Table 1:** Four selected single-nucleotide polymorphisms from three regions

Position	Gene/region	SNP	Ref/minor allele	MAF	*p-*value	Power
127,074,020	PLXNA1-TPRA1	rs11716834	G/A	29	0.0000011	0.959
138,919,221	BPESC1-PISTR1	rs13100625	T/C	15.75%	0.00000039	0.993
173,866,797	NLGN1	rs11927722	A/G	24.45%	0.0000017	0.890
173,986,397	NLGN1	rs549641	G/A	31.33%	0.000009	0.650

**Figure 3:**
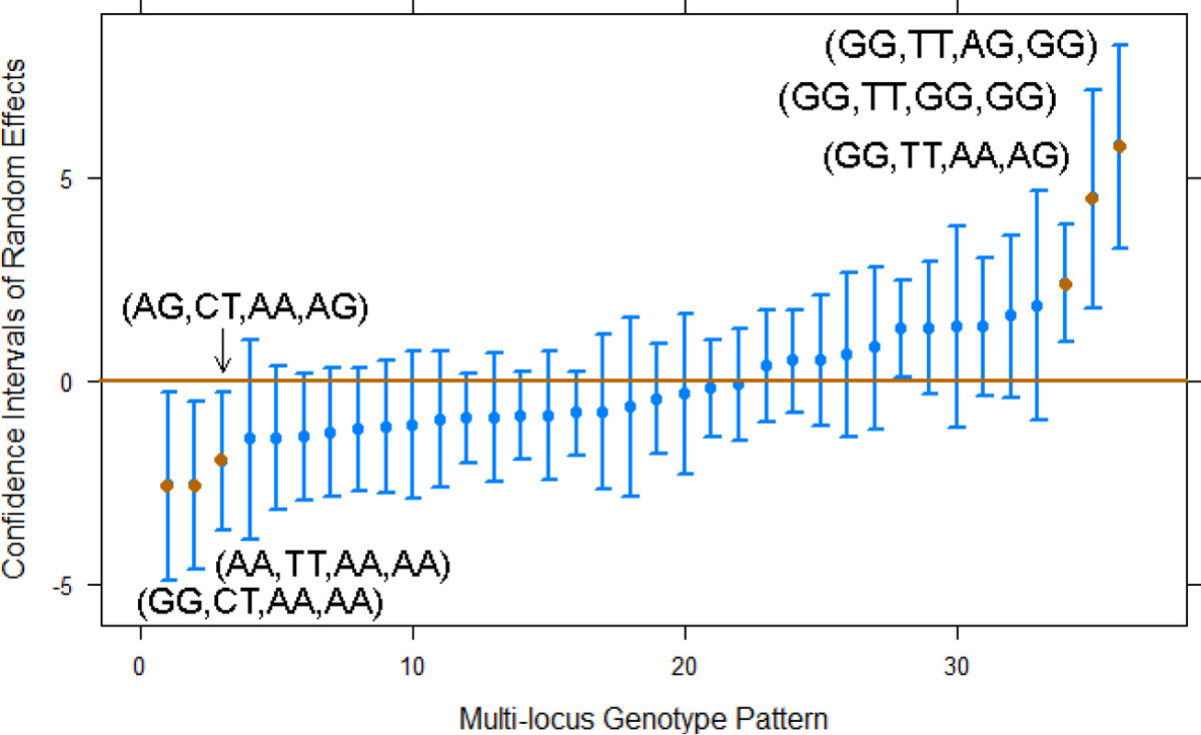
**Predicted 95% confidence intervals (Cis) of random intercepts for the multilocus genotype groups**.

The 860 individuals were assigned to a total of 36 multilocus genotype patterns (patterns with fewerthan six individuals were grouped together). A mixed-effects model with multilocus genotype patterns as random intercept was used to examine the interaction effects among these four SNPs. A parametric bootstrap method with 2000 iterations was then used to calculate the power of detecting significant variance of random intercept on the level of multilocus genotype patterns and to obtain the 95% CIs of the random intercepts for the 36 multilocus genotype patterns.We found significant heterogeneity in SBP trajectories among the multilocus genotype patterns in whichthe 95% CI for the standard deviation of random intercept is (1.79,4.48) with a power of 0.986.Figure 3 shows the 95% CIs of the random intercepts for the multilocus genotype patterns. We found that genotypes (GG,TT,AG,GG), (GG,TT,GG,GG), and (GG,TT,AA,AG) are associated with higher SBP,and (GG,CT,AA,AA), (AA,TT,AA,AA), and (AG,CT,AA,AG) are associated with lower SBP. Our results suggest that there is significant variability in the effect of SBP across the multilocus genotypes.

## Discussion

A linear mixed-effects model was used for GWAS accounting for family structure and time dependence within each individual, adjusting for confounding variables in the first step. Two intergenic regions (*PLXNA1-TPRA1 *and *BPESC1-PISTR1*) and one gene (*NLGN1*) were detected to be significantly associated with SBP after adjusting for multiple testing on chromosome 3.The parametric bootstrap method was used to assess the statistical power of detecting these significant SNPs based on the Bonferroni corrected significance level 0.05/59649 and showed that mixed-effects models provided a powerful tool for longitudinal data analysis accounting for nested data structure, timedependence within repeated measures, and confounding variable. In the second step, the multilocus genotype patterns from four most significant SNPs were used as random effects for the analysis of the joint effect of multiple sequence variants. We found that there was a significant variation in SBP level among these multilocus genotypes. The power analysis strongly supports the evidence of the variation of SBP among these multilocus genotype patterns. The use of multilocus genotypes as random effects in the mixed-effects model framework provides a novel tool for analyzing gene-gene and gene-environment interactions.

Mixed-effects models are a powerful tool with a wide range of applications for longitudinal studies and nested and/or cross-classified data sets. The analysis of large extended-pedigree data using mixed-effects models was discussed in detail by Schork [8]. In practice, if we are interested in both SBP and DBP levels, a multivariate mixed-effects model can be carried out in SAS [9] or R.

## Conclusions

In summary, the analysis of the GAW18 real phenotypes, GWAS, and sequence data allowed us to examine the advantage of a linear mixed-effects model for GWAS and the usefulness ofmultilocus genotypes random effects for joint effects of multiple sequence variants. The novel method proposed could also be developed to identify specific multilocus genotypes that interact with environmental factors for predicting outcomes. Linear mixed-effects models can also accommodate large pedigree data and be extended to multivariate analysis.

## Competing interests

The authors declare that they have no competing interests.

## Authors' contributions

Both authors designed the overall study. YYW conducted statistical analysis and drafted the manuscript. Both authors read and approved the final manuscript.
